# Sustainable livelihood approach with gender-social inclusion perspective for child labor prevention and remediation in rural areas of South Sulawesi, Indonesia

**DOI:** 10.3389/fsoc.2025.1619550

**Published:** 2025-08-11

**Authors:** Idham Irwansyah Idrus, Sopian Tamrin, Riri Amandaria, Muhammad Aksha Wahda, Fitriana

**Affiliations:** ^1^Department of Sociology, Faculty of Social Science and Law, Universitas Negeri Makassar, Makassar, Indonesia; ^2^Department of Guidance and Counseling, Faculty of Education, Universitas Negeri Makassar, Makassar, Indonesia

**Keywords:** child labor, sustainable livelihood approach (SLA), gender social inclusion (GESI), participatory rural appraisal (PRA), social capital, prevention and remediation

## Abstract

Child labor is a global issue commonly found in low- and middle-income countries. A prevention and remediation system based on community knowledge through the Sustainable Livelihood Approach (SLA) with Gender Social Inclusion (GESI) is needed. This study was conducted in three regions with different topographical and geographical characteristics, using qualitative methods and the Participatory Rural Appraisal (PRA) technique, including interviews, focus group discussions, participatory mapping, transects, and seasonal calendars. Findings indicate that child labor is driven by poverty, household characteristics, parents’ education and employment, and limited access to educational services and labor markets. Social, natural, financial, and human capitals serve as strategies for prevention and management of child labor. Utilizing these capitals can be implemented in child labor monitoring and remediation, which can also be conducted as voluntary social audits by community groups. The main recommendation is to establish or assign groups responsible for ensuring the continuity of child labor prevention and intervention efforts.

## 1 Introduction

Child labor is a global issue and a common characteristic of poor and developing countries. Child labor is defined as the illegal employment of children in economic activities (Derby, 2016). Child labor is most prevalent in Africa and the Asia-Pacific Region, where nearly 50% of child laborers are aged 5–11 years (ILO, 2017). Child labor is often found in informal sector industries including agriculture, mining, manufacturing, domestic work, and construction (International Programme on the Elimination of Child Labour, 2011; Avis, 2017). In Indonesia, the case of child labor is no different from that in other developing countries, data from the National Labor Force Survey (Sakernas) conducted by the Central Statistics Agency (BPS) shows this. Sakernas 2020 found that 9 out of 100 children work in the informal sector and 3 out of 4 children are unpaid workers. Child labor, in its worst forms, can be found in various economic sectors such as mining, construction, and agriculture. Sakernas 2022 noted that there were 1.74% or 1.01 million child laborers aged 5–17 years in Indonesia. Provinces with the highest percentage of child labor are mostly in eastern Indonesia, namely in Kalimantan, Sulawesi, Nusa Tenggara, Maluku, and Papua (Al Muflih and Wijaya, 2024).

Agricultural areas located in rural areas absorb a lot of local labor including child labor, where children are vulnerable to various occupational risks, such as fatigue due to long working hours, risk of injury due to the use of tools or machines that are too large for them to handle, physical and mental violence in the workplace, low or no wages. In addition, chemical exposure poses the greatest threat to children’s health (WHO, 2010). And most importantly, the loss of learning time, rest time, and age-appropriate interactions (Keane et al., 2022). Rural agricultural activities are generally informal businesses that lack labor administration and management, making it difficult for the government to monitor.

Poverty is often considered a major factor in child labor (Bharat et al., 2024; Derby, 2016; Gärtner and Gärtner, 2011; Kwofie et al., 2018; Lee et al., 2021; Menon and Rodgers, 2018). Although poverty is the most prevalent cause, child labor is also caused by limited access to social services such as schools and hospitals (Faiz et al., 2024; Sasmal and Guillen, 2015), social norms, family roles, inconsistencies in child labor regulations (Abdullah et al., 2022; Adonteng-Kissi, 2018; Anker, 2000; Babo, 2014; Bansah and Adonteng-Kissi, 2025; Bourdillon and Carothers, 2019; Goto, 2011; Haider, 2008; Hoque, 2024; Kuépié, 2018; Loría, 2020).

Other literature observes that childhood is a time of apprenticeship to learn skills (Omokhodion et al., 2006; Swaminathan, 1998). Parents with a history of child labor in childhood are more likely to place children in child labor (Emerson and Souza, 2003; Khan et al., 2024; Huamaní-Huapaya and Raúl, 2021). Other literature analyzes the impact of agricultural exports on decreasing school enrollment and increasing child labor (Lin, 2021).

Using the sustainable livelihood approach framework with gender and social inclusion approach. SLA is a framework used to understand the complexity and multidimensionality of livelihoods. SLA works by linking various livelihood contexts with livelihood resources and strategies. An important element in SLA is the analysis of institutional processes and organizational structures, as they mediate the use of livelihood resources with livelihood strategies (Scoones, 2015). There are six dimensions analyzed in SLA, namely context conditions and trends, livelihood resources, institutional processes and organizational structures, livelihood strategies, and sustainable livelihood outcomes. The Gender and Social Inclusion (GESI) perspective is a paradigm used to understand the meaning of women and men as well as marginalized groups about living space and social relations related to the regulation and control of living space and livelihoods (Anggia and Abdulkadir-sunito, 2023).

This study aims to determine (1) the factors causing child labor in rural areas of South Sulawesi; (2) the structure of community knowledge about children and its implications for the social value of children in rural areas of South Sulawesi; and (3) the form of utilization of livelihood resources as a strategy in the prevention and remediation of child labor in rural areas of South Sulawesi. The results of this study are expected to produce a recommendation as a system for the prevention and remediation of child labor.

## 2 Sustainable livelihood approach with gender and social inclusion approach

The Sustainable Livelihood Approach (SLA) is a framework for understanding and improving people’s lives, especially marginalized communities through sustainable resource utilization. SLA was first introduced by Robet Chamber and Conway and popularized by the Department for International Development (DFID) (Chambers and Conway, 1992). SLA is an approach that observes livelihoods as the ability of individuals and households to meet basic needs in a sustainable manner, including when experiencing shocks or stresses.

SLA views livelihoods as spatially complex, multidimensional, spatially and temporally diverse, and socially differentiated. Influenced by many factors from local conditions to structural political economy processes. In this section, SLA as an analytical framework allows it to be used to understand this complexity and analyze the relationship between existing contexts or components.

**Figure 1:**
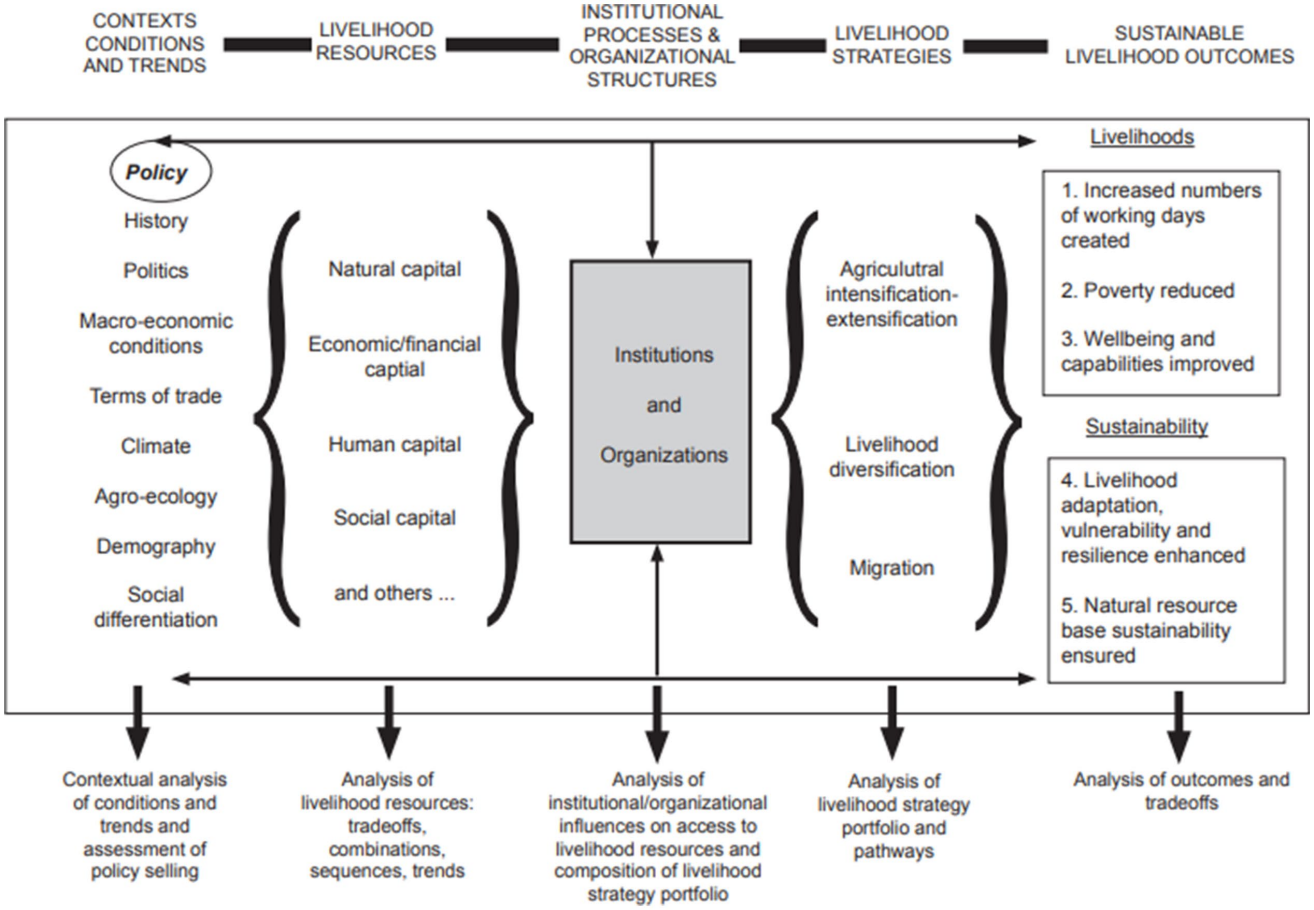
Sustainable livelihood approach (Scoones, 2015).

The SLA framework consists of the context of conditions and trends, which is a contextual examination of various conditions including history, policies, politics, macroeconomic conditions, demographics, climate, and social differentiation. The second is the analysis of livelihood resources, which includes natural capital, economic capital, human capital, and social capital. The third is the livelihood strategy, which is an analysis of the utilization of livelihood resources that yields results for livelihood sustainability. Among the livelihood resources, *institutional processes* are attached, which serve as a link between the livelihood resources. The institutional process explains how social institutions and organizations bridge livelihood strategies and resources. Livelihood strategies and resources are in a complex and interrelated social relationship. Livelihood strategies and resources meet in sociocultural and political dimensions that can explain how and why asset inputs are linked to livelihood strategies and outcomes. Livelihood strategies and resources are influenced by power and politics, which include access, rights and governance (Scoones, 2015) (Figure 1).

The GESI perspective in SLA is an approach tool that analyzes and understands the meaning of women, men and marginalized groups about living space and the various components within it that are the source of livelihoods both at the individual and group (household) levels (Abdulkadir-Sunito et al., 2019). In addition, GESI also analyzes who and how women, men and marginalized groups are involved in the process of governance and control of living space and livelihoods.

## 3 Data collection methods and analysis techniques

This research is a qualitative study that uses the SLA with a GESI perspective. SLA is a framework used to understand the complexity and multidimensionality of livelihoods. SLA works by linking various livelihood contexts with livelihood resources and strategies. An important element in SLA is the analysis of institutional processes and organizational structures, as they mediate the use of livelihood resources with livelihood strategies (Scoones, 2015). There are six dimensions analyzed in SLA, namely context conditions and trends, livelihood resources, institutional processes and organizational structures, livelihood strategies, and sustainable livelihood outcomes. The GESI perspective is a paradigm used to understand the meaning of women and men as well as marginalized groups about living space and social relations related to the regulation and control of living space and livelihoods (Abdulkadir-Sunito et al., 2019).

Bourdieu’s social reproduction theory and poverty trap theory are used to understand the phenomenon of child labor persistence. The theory explains how social status, social inequality is maintained and passed on from generation to generation (Bourdieu, 1990). The main proposition of Bourdieu’s social reproduction is that the conversion of economic capital into cultural capital is a strategy to maintain or improve one’s position in the social space; secondly, habitus mediates the conversion of economic capital into cultural capital; thirdly, capital and terrain are interrelated because capital has no existence without terrain (Farid et al., 2021). Poverty traps are defined as self-reinforcing mechanisms that cause poverty to persist (Barbier and Hochard, 2019), such as low knowledge and competence, financial limitations and lack of access to capital.

The research was conducted in three areas with different characteristics, both topographically and geographically, namely in Kanreapia Village which is a mountainous area, Samangki Village which is a lowland area, and Laikang Village which is a coastal area. The research began with secondary data collection to analyze population and agricultural data for the last 3 years. Primary data collection was conducted through Participatory Rural Appraisal (PRA) techniques. The PRA technique was used with the aim of exploring local perspectives, values, social dynamics related to the issue of child labor. We understand that PRA is not just an instrument for gathering information, through this research we encourage changes in community perspectives on child labor through active community participation in research activities. In this research, PRA is used to analyze existing livelihood sources, how they are utilized, and how institutions and institutions affect their utilization. The information obtained will be linked with data and information on child labor.

The PRA stages consist of interviews, focus group discussions, participatory mapping, transects, and seasonal calendar preparation. Interviews are conducted to explore informants’ life experiences in the utilization of livelihood resources, control and governance of livelihood resources, social norms, childcare, and to explore knowledge on child labor practices. *Focus group discussions* (FGDs) were conducted twice in each region. The subjects of the FGD consisted of stakeholders and women’s groups, which were conducted separately.

Participatory mapping was conducted by making women and marginalized groups the subjects. They were asked to explain about who has access to, controls, regulates the utilization of livelihood resources, and how the process works. In this section, informants also explain how institutions and social institutions play a role in the management and access mechanism to livelihood resources. Transects generate data on topography, land use, vegetation, and land ownership structure. The transect was conducted by the researcher by walking around the research location. Preparation of a seasonal calendar, generating information on the community’s agricultural activities in one season. The preparation of the seasonal calendar was conducted in a participatory manner through a meeting by presenting women’s groups, marginalized groups and stakeholders.

Informants in this study were women, stakeholders and marginalized groups. The selection of women as informants was based on the GESI approach, which aims to obtain women’s perspectives. In rural life, women play an important role in household affairs, namely as financial managers, caregivers, and often get gender-based injustices such as discrimination in the fulfillment of basic rights, management and control of resources.

Informants from marginalized groups and stakeholders aim to provide an explanation of how they are involved in regulating and utilizing livelihood resources. There were 35 informants in this study, including farmers and tenant farmers, women and housewives, and stakeholders. Their voices are important to illustrate how the regulation and utilization of livelihood resources impact livelihood strategies and the sustainability of these livelihoods. The initial assumption of this research is that child labor is a rational choice and a livelihood strategy.

The data analysis used is descriptive data analysis. After data and information were collected, classification and review were conducted to analyze the interrelationship between field findings. To ensure the quality of data and information, triangulation was conducted. Informants in this study were selected based on categories including farming and fishing communities, government officials, community leaders, and children. Women, children and marginalized groups were the main informants in this study.

## 4 SLA result description

### 4.1 Living space analysis

The analysis of living space is an analysis of the living space for the community. Living space includes humans, other living things (animals and plants), abiotic elements, and socio-economic, political and cultural elements. The analysis of living space in this research uses the method of sketching the village through *transect* activities, tracing the history of the village, observation and interviews.

The living space identification data is presented as follows (Table 1).

**Table 1:** Data from the identification of living spaces in the 3 research villages.

Living space	Leading commodities	Who accesses	Master	Ownership
Kanreapia Village, Tombolo Pao Sub-District, Gowa Regency				
Land	Carrots, cabbage, chicory, potatoes, leeks	Male and female	Male and female	Individual/family
River	Water	Male and female	Male and female	Communal
Groundwater	Water	Male and female	Male and female	Individual/family
Forest	Wood, groundwater	Male and female	Ministry of forestry	Government
Samangki village, Simbang sub-district, Bantimurung sub-district				
River	Water	Male and female	Male and female	Communal
Forest	Wood, palm, honey	Male and female	Bantimurung - Bulusaraung National Park	Bantimurung - Bulusaraung National Park
Garden	Beans, corn	Male and female	Male and female	Individual/family
Spring water	Water	Male and female	Male and female	Communal
Sawah	Rice, peanuts	Male and female	Male and female	Individual/family
Laikang Village, Mangara Bombang Sub-district, Takalar Regency				
Sea	Lawi-Lawi (Gulfweed) Tude/Kerang (Shells) Fish Shrimp Crab Taripang (Sea cucumber) Agar (Seaweed)	Male and female	Male and female	Communal
Land/rice field/garden	Corn Green Beans Mango Sukun Coconut Rice Livestock (Cattle, Chicken, and Goat)	Male and female	Male and female	Individual/family
Estuary	Fish Crab Shrimp	Male	Male	Communal
Empang	Shrimp Lawi-Lawi Bolu Fish	Male and female	Male	Individual/family
Page	Banana Tree Sweet Potato/Cassava Eggplant Small/large Lombok Moringa Lime Lemongrass Pumpkin Pineapple Spinach Kale Watermelon Citronella leaf Mango Water guava	Male and female	Male and female	Individual/family

Research data (processed).

The data in the table above was obtained through various methods such as *transect*, FGD, and observation. The identification of living space will facilitate the analysis of community livelihood sources. The assumption is that community livelihoods cannot be separated from living space. Furthermore, to understand the relationship between the community and the living space, the mechanism of access and control, it is explored using interview techniques.

Living space contains socio-economic-political-cultural-spiritual meanings. The three research locations interpret water and land as an integral part of their lives. Land and water are tools to achieve welfare, markers in social stratification and a means of spirituality. From the transect results, it was found that the use of living space is dominant for settlements and agricultural and fishery activities. Land is used for farming, gardening, searching for forest products such as wood. While the water area is used for seaweed farming and shrimp ponds for the people of Laikkang village, and fishing.

Social meanings relate to access, i.e., who controls and how roles are divided between men and women. The control of living space is dominated by men although access remains open to women. In Laikang Village, ownership and control of the seaweed cultivation area is in the hands of men. Women are only involved in the activities of planting and drying seaweed, as family workers or as wage laborers. Similarly, in Kanreapia and Samangki Villages, inheritance rights over agricultural land are generally given to sons, and women receive residential houses. Some of the girls also received small plots of agricultural land. None of the informants owned more than two hectares of land. Some of them do not even own land so they have to work as sharecroppers. We found it difficult to obtain detailed information on land tenure. This is because it is a sensitive issue. We obtained information about land tenure through interviews with various parties who know information about land ownership such as collectors, tenant farmers, and village officials.

Economically, living space supports the community’s livelihood. Women and (especially) children involved in labor often receive lower wages than men. In seaweed farming, women are paid based on their work, for example, tying seaweed seedlings on ropes will be paid according to the number of ropes. For agricultural and plantation activities in Samangki and Kanreapia Villages, women become daily farm laborers at harvest time and some help their husbands in managing their farming areas. From a political perspective, the control of living space reflects power relations. Local elites or corporations can influence the control of living space. In the seaweed farming area in Laikang Village, the regulation on the control of the cultivation area is regulated in the regulations issued by the village government, at the district level it is regulated by the Regional Regulation of Takalah Regency Number 138 of 2013 concerning the Determination of Seaweed Industrial Area in Punaga Village, Mangarabombang District.

Community culture also shapes the meaning of living space. Rivers or springs may have sacred values in local traditions. As a form of gratitude and appreciation for land and water, the Kanreapia village community conducts various activities such as *Kamisi’* and thanksgiving ceremonies every year. *Kamisi’* is a mutual cooperation activity to clean the water every Thursday, and thanksgiving is done once a year as a form of gratitude for the abundant harvest. In Samangki and Laikang villages, harvest parties are held as an expression of gratitude and thanks to nature.

### 4.2 Livelihood analysis

Livelihood analysis is an analysis of capital or assets including human capital, natural capital, economic capital, social capital, and physical capital (Li et al., 2020; Morse, 2025; Nath et al., 2020; Su et al., 2021).

#### 4.2.1 Human capital

From a household perspective, human capital includes skills, knowledge and physical abilities that can be used by households. Skills and physical abilities can be used for productive work. Skills include agricultural techniques, fisheries both capture and cultivation, and other skills outside of agriculture and fisheries. In agriculture such as in rice fields and gardens, the main skills include pre-planting to harvesting activities. For example, skills in seed preparation, fertilization, maintenance and harvesting. From all informants, farming skills are acquired through a process of learning and habituation since childhood. Skills are an integral part of physical abilities. Other skills besides agriculture are skills in construction. They work as masons or carpenters. Those who have no skills and rely on their physical abilities only work as laborers.

Knowledge in human capital plays a very important role because it can be a link with other components. Knowledge has a contribution to determine the pattern of care for children, the value of children. Parenting relates to parents’ or caregivers’ knowledge about the protection and fulfillment of children’s rights. For example, fulfillment of nutrition, safe situations for children, fulfillment of children’s rights to learn and play. From the results of interviews and FGDs, it was found that parents understand how to fulfill nutrition for children, but the education aspect is generally not a top priority in fulfilling children’s rights. The expensive cost of education is the most frequently cited reason. We found that the average education of parents was only primary and junior secondary school. In addition, child marriage is also a factor in stopping children’s education.

To ensure the protection of children, parents bring their children with them to the workplace (FGD in Kanreapia village) and some leave them with relatives. Some informants said that bringing children to the farm is a way of introducing them to their parents’ work. Children who have sufficient physical strength have been involved in work such as cleaning the garden or becoming daily farm laborers during school holidays.

#### 4.2.2 Natural capital

Natural capital is the availability of natural resources that can be accessed by households such as land for gardens or rice fields, forests, rivers, lakes, swamps, estuaries and the sea. Most households in the three research villages own land used for housing and agriculture, ponds, and areas for seaweed cultivation. There are several household members who manage rice fields or gardens owned by others with a profit-sharing system and some of them become farm laborers. The involvement of women and children in agricultural and plantation areas is quite large, especially in maintenance activities such as watering and cleaning from weeds and harvesting. Weather conditions such as the dry season in Samangki and Kanreapia, and the long rainy season in Laikan are challenging. Agricultural activities are particularly vulnerable to crop failure, or increased operational costs. This encourages farmers including children to seek more secure income channels, for example by becoming construction workers.

**Figure 2:**
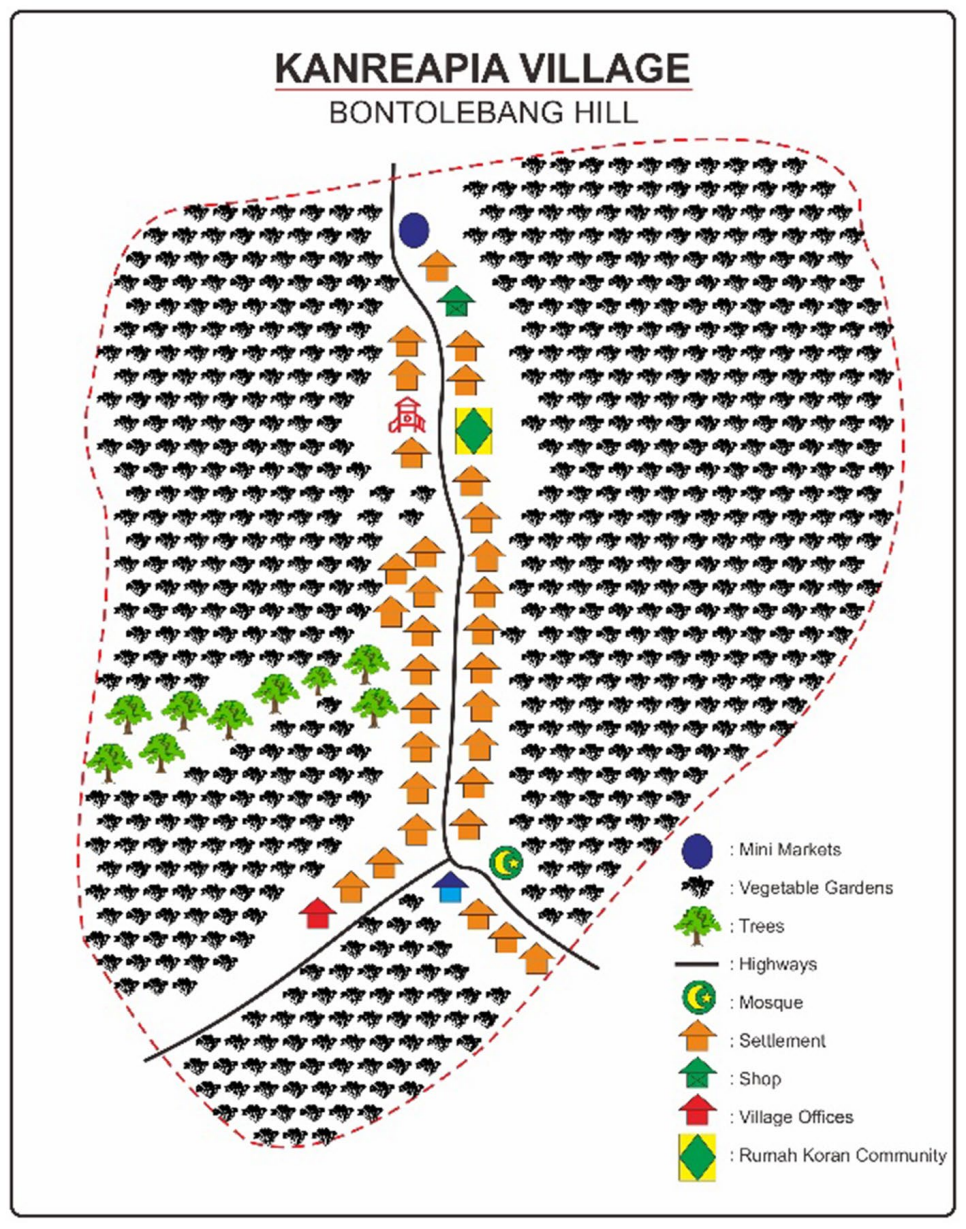
Sketch of living space in Bontolebang Hamlet, Kanreapia Village. Source: Research data (processed).

To identify the components present within the living space that constitute natural capital, a transect is conducted. This activity produces data in the form of a sketch of the living space, as exemplified by the transect results in Bontolebang Hamlet, Kanreapia Village, shown below (Figure 2).

#### 4.2.3 Economic capital

Economic or financial capital is access to finance such as income streams, savings, and loans. Apart from agriculture, Farmers generally have several sources of income, such as wages from construction work, trading, and other services. From agricultural activities, income diversification is obtained from crop diversification. For example, in Kanreapia apart from growing carrots and cabbage, farmers also grow leeks, celery as short-term crops worked by women and children.

From the FGD results, men and women have equal access to financial resources. Although women are given the task of saving, the utilization is based on mutual agreement. The sale of vegetables, peanuts, grain, and seaweed is done by men and then the proceeds are saved by women. Household expenditure consists of daily consumption expenditure, children’s school fees, medical expenses, and farm operational costs. In difficult financial conditions, women take the initiative to seek loans from their closest relatives. Larger loans are usually obtained from cooperatives or banks.

#### 4.2.4 Social capital

Social capital is a social resource that can be utilized such as solidarity, a sense of kinship, and trust. In agricultural activities farmers need the labor of others in various jobs. To reduce expenses, farmers usually form groups and help each other as a form of solidarity. In all three villages, farmers will ask relatives or neighbors to help at harvest time. It is reciprocal and not paid with wages, but mutual help in every harvest activity.

In Samangki and Kanreapia villages, social capital was found during the dry season when they fairly distributed water for communal consumption. In Samangki village, solidarity was strengthened when they were involved in an agrarian conflict with the Bantimurung Bulusaraung National Park management. In Laikang village, social capital is evident in the activity of tying seaweed seeds on ropes. Landowners will employ their closest neighbors and provide adequate wages. One landowner said that he would prioritize women who did not have permanent jobs.

#### 4.2.5 Physical capital

Physical capital includes supporting infrastructure such as roads, education facilities, health facilities. From the FGD results, the availability of school facilities is one of the causes of the decision to stop attending school. From the three regions, education facilities only reach the junior high school level. Senior high schools are only available in the capital city of the kecamatan or kabupaten. Children who do not have a vehicle are likely to choose to stop going to school. Health facilities are generally available at the village health post (Poskesdes) and sub-health center (Puskesmas Pembantu).

### 4.3 Analysis of social relations, institutions and organizations related to livelihood resources

The analysis of social relations, institutions and organizations is a section that will examine how institutional processes, including institutions and organizations (formal and informal), mediate livelihood strategies toward livelihood access. Social institutions and organizations are instruments in regulating livelihoods in various dimensions in the setting of social relations such as gender, social class, religion, and ethnicity.

Analysis of institutions and organizations is crucial in understanding the process of access to livelihood resources and livelihood strategies. For example, whether there are access restrictions related to gender, age, ethnicity, marital status, disability, and social class (Anggia and Abdulkadir-sunito, 2023). No restrictions on access to resources were found in any of the three areas. Both women, men and children have equal access to family and communal resources. A case in point is water management for agricultural activities and consumption. Both Samangki and Kanreapia villages are managed independently and under the supervision of the local village government. No restrictions on water distribution were found.

However, in the case of agrarian conflict between the Samangki village community and the Bantimurung Bulusaraung National Park management, there are restrictions on the community in utilizing forest products such as firewood, sap water, and forest honey. In Laikang Village, the village government has the control to regulate the control of seaweed cultivation. The aim is to prevent land conflicts. In relation to child labor or the decision to employ children, there are no restrictive institutions. Limited parental income, asset ownership, and obligations after marriage (child marriage) are push factors for children to engage in child labor. What we found was normalized by social institutions.

### 4.4 Institutional analysis of social relations that influence child labor

The research found a variety of institutions in the three regions as shown in Table 2.

**Table 2:** Social institutions in the study area.

No.	Name of institution	Type	Who can access	Function and role
1.	LPM	Formal	Male and female	Planning, implementation and supervision of village development
2.	Farmer Group	Informal	Male and female	A medium for coordination, cooperation, and exchange of ideas between farmers
3.	Youth Organization	Formal	Male and female, 16–30 years old	Youth Organization
4.	Pokdarwis	Formal	Male and female	Tourism management
5.	Posyandu	Formal	Female	Baby, toddler health
6.	Dasawisma	Formal	Female	Helping the PKK program work
7.	NGO	Formal	Male, female	Monitoring
8.	Ta′lim Assembly	Informal	Female	Religious studies
9.	Youth/Women move forward together	Formal	Male, female	Youth organizations
10.	U16 Village Team	Informal	Male	Football club
11.	Cultivation Group	Formal	Male, female	Fish Farming Farmer Group Waste Bank Fisheries and Capture Fishermen Group (sikatutuiki)
12.	Customary Institutions	Formal/informal	Male, female	Manage customary affairs

Research data (processed).

Institutions that have been identified can be classified into:

1. Group by livelihood
2. Youth group
3. Women-based groups
4. Groups formed from government initiatives

In child labor prevention and handling activities, these groups can function according to their respective roles. The group has the ability to regulate the behavior of its members so that it can be modified for the purpose of preventing and handling child labor. Customary groups/customary institutions have the power to act as social control through well-institutionalized rules. For example, rules on area management rights, agricultural production sharing, marriage matters.

In Kanreapia Village, a customary institution was established, one of whose functions is to regulate the marriage system and conflict resolution. In terms of marriage, customary institutions are the institutions that regulate marriage procedures, especially in siri marriages, including child marriages. The same applies to the divorce process in siri marriages. This institution has succeeded in reducing conflicts due to betel marriage and divorce. In Laikang Village, one of the functions of customary institutions is to regulate control over marine areas for seaweed cultivation activities. With a set of rules that have been established, customary institutions in Laikang village have succeeded in minimizing the potential for land conflicts between farmers.

### 4.5 Livelihood strategy analysis

Livelihood strategies are understood as household efforts to continue their lives and livelihoods at a safe or stable status. Livelihood strategies in rural communities fall into two categories: natural resource-based and non-natural resource-based livelihoods. Based on the assumption that the main cause of child labor is poverty, it is important to analyze livelihood strategies at the household level.

In this section, the question is asked, do households engage in agricultural and off-farm livelihood strategies, both within and outside the village?

In Laikang Village, livelihood strategies are generally related to agriculture and fisheries, especially seaweed farming. According to information from one of the village officials, before seaweed farming the community’s income was very low and unemployment was high. This was due to the geographical conditions that did not support agricultural activities. After seaweed farming began to be pursued by the community, the community’s livelihood slowly improved and absorbed labor, thus reducing the unemployment rate.

Seaweed farming is not as easy as one might think, with challenges such as weather and uncertain prices. To maximize income, various strategies are used, such as selecting seeds according to weather and climate conditions, expanding land, collecting harvests and selling them if prices are high. If prices fall, labor is reduced. Expanding the land will be followed by additional labor from other households.

To maximize yields, almost all family members are involved with various divisions of labor. In seaweed farming, men are given heavy work such as installing seedlings, harvesting seaweed. Meanwhile, women and children work to attach seaweed seeds to ropes, and separate harvested seaweed. Some communities with no land or little land have livelihood strategies that are not based on natural resources. For example, working in the city as a trader, working as a carpenter and builder, and working as a food seller.

In Kanreapia Village, the main occupation is in the agricultural sector. Previously, the community of Kanreapia village farmed with chayote commodities, but the results were not as good as the current commodities such as chicory, cabbage, leeks, and carrots. Nowadays, with a variety of agricultural commodities, the community’s income is getting better. Apart from the agricultural sector, livelihood strategies are also directed at non-natural resource activities. Off-farm livelihood strategies are carried out when agricultural activities do not provide sufficient yields, for example during the dry season when there is a threat of crop failure. Some families do this if there is an opportunity. Activities undertaken include becoming a trader, working in the construction sector, and other service sectors.

To maximize family income, all family members are involved in productive activities. Family members are involved in agricultural activities, but with adjusted workloads. Children usually help to clean the garden and harvest the crops. Adults, on the other hand, do the heavy and long-term activities, such as planting seeds, managing irrigation, and fertilizing. During the harvest season, smallholders are used as hired farm laborers. All (productive) family members are involved in these activities.

In Samangki Village, the main livelihood strategy is in the agricultural sector: rice, corn and secondary crops in the dry season. However, there are some households that strategize by working in the city and migrating to Java and Kalimantan. Since the land conflict with the Bantimurung Bulusaraung National Park, livelihood strategies outside of natural resource utilization have been increasingly employed. Limited access to forests and productive lands has forced the community to shift their livelihood strategies to other channels, especially to non-agricultural activities, such as becoming traders and working in cities.

### 4.6 Contextual analysis of conditions, trends and policies

In this section, the conditions of the region, including its history, politics, and vulnerability, will be studied. Trend analysis is related to the population picture, livelihoods, and changes caused by new technology.

#### 4.6.1 Laikang village

Based on the narrative of the Village Head, Laikang was once a kingdom whose founders were scholars or propagators of Islam. Sayyid Jalaluddin was a highly respected figure and was the founder of the Cikoang kingdom and was continued by his son named Sayyid Umar Al Aidid. The teachings of Sayyid Jalaluddin are still continued to this day, namely *Maudu lompoa.*

Administratively, Laikang village is a village in Laikang sub-district, Takalar district. Laikang village has six hamlets, namely Puntondo, Laikang, Turikale, Ongkowa, Boddia, and Pandala. The area is about 19.6 square kilometers. The total population in 2022 in Laikang Village is 5,969 people consisting of 2,938 men and 3,031 women. In 2021, the population of Laikang village was 5,849 people, consisting of 2,876 men and 2,973 women. Analysis of the political situation shows that Laikang Village has an important position. In the 2024 General Election, Laikang Village was eyed by many politicians because of the large number of voters. However, this condition does not create a gap, it can be said that Laikang villagers are quite mature in politics, respect each other’s political choices, and do not make differences in choices a tool for division. From the results of our analysis during the research, no political power dominates in Laikang Village.

**In terms of vulnerability analysis**, Laikang Village is very vulnerable to drought. Many residents’ wells have dried up, so to fulfill their daily water needs they have to buy water at a price of Rp. 200,000 for each cubic meter of water. In a week each household needs at least 2 cubic. This situation greatly affects community finances in Laikang village.

#### 4.6.2 Kanreapia village

The word Kanreapia comes from two words, Kanre and fire, which means burning and fire. According to the story that developed in the community, Kanreapia village once experienced a great fire, from that event the name Kanreapia was immortalized as the name of a village. Administratively, Kanreapia village was officially formed in 1990. The village head changed and was led by H. Rusli. H. Rusli as the village head has a strong influence. With the village development program implemented, he was able to attract sympathy and support from the community.

#### 4.6.3 Regional vulnerability analysis

Located at the foot of Mount Bawakaraeng, Kanreapia village has cool temperatures that are suitable for agricultural activities. However, along with the development of agriculture, the threat of environmental damage also continues to occur. The conversion of forest areas into agricultural areas has the potential to cause landslides and reduce water sources.

During our research in Kanreapia Village, we also found many cases of child marriage, especially among girls. In a year, there is usually 1 case of child marriage or even more. We explored the reasons for this through FGDs with housewives, and found that child marriage is a common occurrence. Some of the FGD participants were also married as children. Tradition and poverty were the most commonly cited reasons. Girls tend to be married off early to reduce the burden on the family (for poor families), and for farming families marrying off girls is a way to maintain their agricultural assets.

The high rate of child marriage has an impact on the low level of education in the community, with the average finishing only junior high school. After marriage, children will be confronted with the adult world such as taking care of the household, earning a living, and various other activities carried out by adults. Child marriage is a concern for the village government, one of the efforts made is to encourage children to stay in school up to university level. Providing support in the form of learning and sports facilities. Another step taken is to encourage community welfare through PKK activities, agricultural counseling, and facilitation of social assistance for poor families.

Child marriage is understood as a violation of children’s rights, so child marriage cannot be facilitated by the state. Child marriages that occur are generally conducted in betrothal or unregistered marriages. Child marriage is difficult to prevent, especially after the proposal process takes place. One dusun head recounted that an *elopement* had occurred because the couple who proposed marriage did not obtain permission from the government.

To minimize the impact of child marriage, the community agreed to establish a customary institution to mediate the siri marriage process. Through this institution, siri marriages (including child marriages) that occur can be monitored properly. Including if a divorce occurs, it must be mediated by the customary institution.

Child marriage that occurs is in a persistent status and takes place between generations. The average education of parents is only up to junior high school, because they were married off as children. On average, children who perform child marriage come from child marriage households. This has become a tradition and is considered a natural thing, so child marriage takes place between generations.

#### 4.6.4 Samangki village

Samangki Village was originally located in Simbang District (before it became a sub-district), which later changed its name to Bantimurung Sub-district. In 1999, Kecamatan Bantimurung was divided into 2 (two) sub-districts, namely Kecamatan Simbang (Definitive in 2001) and Kecamatan Bantimurung itself.

The aforementioned sub-district expansion positioned Samangki Village within the Simbang Sub-district area until now.

Before becoming a definitive village, the word Samangki (Bugis language means we arrived together) or in Makassar language is also called Samanggi (a’samangki), the conclusion is that the pronunciation of the word Samangki is pronounced by the Bugis tribe while the pronunciation of Samanggi by the Makassar tribe, at first Samangki Village was part of Jenetaesa Village which at that time still had the status of Rukun Kampung (RK), namely RK Samanggi, Due to the development of the region, the development of the population and the development of the need for community services, in 1989 Jenetaesa Village was divided into 2 villages, namely Jenetaesa Village as the Main Village, while Samangki Village as the Expansion Village, and in 1991 Samangki Village officially became a Definitive Village led by **H. Husain** in 1991–2007, namely 2 villages**. Husain** in 1991–2007, namely 2 periods, and in 2007 the elected village head election was **H. Makmur. HS** as Village Head for the period 2007–2013, and re-election of the village head in 2013 re-elected **H. Makmur. HS** as Village Head for the period 2013–2019. Then in 2019 a village head election was held which elected **Hj. Darwana, S. Pd** as Village Head for the period 2019–2025.

## 5 Discussion

### 5.1 Factors causing child labor in rural areas of South Sulawesi

Child labor is a social construction that is conceptualized variously through cultural practices and social institutions that have gone through a long historical trajectory. The diverse conceptualizations of child labor have led to a variety of causes of child labor. The case in Europe before the 20th century saw an increase in demand for cheap labor after the industrial revolution. Many children were employed for long periods of time and heavy workloads. These children came from poor families and were paid less than adult workers.

Poverty is not the only cause of child labor, but it is the most common factor in many cases of child labor in Asia and Africa. Child labor can also arise from structural and cultural factors. Structural factors include failure of the education system, malfunctioning national employment system, labor market demand, urbanization, economic and political instability. Cultural factors include the culture of local communities that legalize child labor. Recent climate change also has an impact on the vulnerability of children in their activities as child laborers.

Using an economic approach to analyze the causes of child labor. The economic approach sees a correlation between poverty and child labor. Poor families will always choose to encourage their children to engage in labor, usually as manual laborers on farms, and selling services in the informal sector. In the household context this is income diversification. On the other hand, poverty and economic deprivation do not motivate parents to engage their children in work. Land ownership is seen as more correlated with the decision to employ children (Rogers and Swinnerton, 2007). Families who own large tracts of land despite being economically well-off will rely heavily on the labor of their children. The decision is legalized by social norms that allow children to work for their parents. Girls are also potentially harmed by this practice. Girls will be given the task of taking care of the household such as cooking, cleaning, and taking care of their young siblings. Both her parents and her brothers work outside the home. This certainly robs them of their childhood to play, learn, and socialize with other children.

In traditional or farming families, children are shaped according to the expectations of the community or long-standing traditions. The process takes place within the family because the family is the first and main socialization medium for children. Girls will be shaped in feminine ways, they are taught about nurturing, cooking, cleaning the house, and entertaining guests. While boys are molded to be masculine, since childhood they have been introduced to various jobs outside the home, and completing heavy tasks. For example, plowing rice fields, cleaning gardens, taking care of livestock, and so on. In agrarian societies, parents’ orientation in shaping their children is fully geared toward the continuity of the family business. Although this has changed, it can still be found in some areas.

There is an interconnected relationship between the causes of child labor, which in turn contributes to other child problems. For example, children who are involved in child labor will lose time to attend school/get an education so that they do not have enough skills to enter the workforce (Lee et al., 2021). This problem will lead to poverty. Conversely, children who drop out of school do not have many life choices, especially those from poor families. So working is the main choice. Here we can understand how the impact of the failure of the education system to reduce the rate of child labor cases.

A study in the agricultural regions of Ghana found that cultural practices play an important role in children’s involvement in agriculture in rural areas such as child marriage. Any children who marry (child marriage) will have the responsibility to take care of the household. Boys will be responsible for earning the family’s livelihood, while girls will be responsible for taking care of the home. Both are child laborers, either paid or as child laborers in the domestic sector. They miss out on childhood activities such as playing, learning, and being cared for by their parents. Cases in Kanreapia and Samangki villages show that married children will be involved in various adult jobs.

In terms of socialization, children accompany parents to the farm to work, which is a way of passing on skills to children (Adonteng-Kissi, 2018). In this part, parents involve children in hazardous work and work with children for a long duration of time, so that children lose time to play, study, get enough rest. Similarly, in the three research areas, work for children is a form of devotion to their parents, a form of repayment, and as preparation for their future. Therefore, working for children is something that is commonplace, and becomes different if children are not involved in their parents’ work. In regulations governing child labor, there are several concepts such as working children and child laborers. Working children are those who are involved in their parents’ work in accordance with their portion as children and do not endanger their physical and mental health. Meanwhile, child laborers are those who work outside their capacity as children and endanger their physical and mental health. This is not well understood. There is no prohibition for children to work as long as they pay attention to safety aspects and fulfillment of children’s rights.

In Indonesia, child labor is defined as children working below the minimum age allowed to work. The Indonesian government’s attention to the issue of child labor became serious after the 1997 economic crisis. Through Law No. 20 of 1999 and Law No. 1 of 2000, the government ratified ILO Convention No. 138 concerning the minimum age for employment and Convention No. 182 concerning the prohibition and immediate elimination of the worst forms of child labor. Within the regulatory framework, the minimum age at which children can work is 13 years old to do light work as long as it does not interfere with physical, mental and social development and health. For 15 years old to before 18 years old are allowed to do regular work for a maximum of 8 h per day and 40 h per week (Table 3).

**Table 3:** Age limits of child labor in the Indonesian legal framework.

	Type of work			
Child’s age	Occupations that are exempt from the minimum age requirement	Light work (maximum 3 h per day and 15 h per week)	Regular work that does not cause harm (maximum 8 h per day and 40 h per week)	Worst forms of child labor (including child endangerment work)
<13 years	Not child labor	Child labor	Child labor	Child labor
13–14 years old	Not child labor	Not child labor	Child labor	Child labor
15–17 years old (young worker)	Not child labor	Not child labor	Not child labor	Child labor

Secondary data (processed).

Child labor in the agricultural sector is at least faced with threats to safety, health, welfare, and the non-fulfillment of several children’s rights. Threats to safety are mainly for those in hazardous work situations, such as children’s involvement in the use of pesticides and other chemicals, children’s involvement in the transportation of crops, and the use of sharp objects in agricultural activities, as well as children who are forced to work for long periods of time (Hurst, 2022). Long working hours cause children to lose time to attend school, play, and socialize according to their age. The causes of child labor do not stand alone as a problem. The causes are interrelated with one another, if described the causes of child labor is a circle of problems that continues to rotate.

### 5.2 The structure of community knowledge about children and its implications for children’s social value in rural South Sulawesi

People’s knowledge or conceptions of childhood influence a child’s social value. In this study, knowledge about childhood was explored through interview techniques, hope tree analysis and risk. In all three areas, children are understood as the successors of their parents and must be prepared from an early age. Children who grow up in farming families will generally be prepared to become farmers. From an early age, they are introduced to various agricultural activities.

Almost all parents hope that their children will have a better life. Success as a businessman, a policeman, a trader, and a civil servant. But they realize that what they dream of is not easy to achieve, there are challenges they face. For example, the high cost of education, limited education services, socio-cultural background and children’s low motivation to go to school. The cost factor is the dominant barrier. Children from poor families will decide or be forced to stop going to school and decide to work.

From the three research locations, several understandings of childhood were found. First, understanding children as family members who have an obligation to be devoted to their parents. For low-income households, devotion is synonymous with productive work to help the family’s finances. Therefore, the choice to work is something that is expected. Second, understanding children as the successors and heirs of the efforts made by parents. Farming households have been preparing their children for a future as farmers since childhood. Based on their age, children have been assigned tasks to take care of the farm. For example, in Kanreapia, if children are 16 years old, they are already given the responsibility to manage a patch of land.

Stegemen argues that childhood in the perception of developed countries is a time for children to grow and develop, to get an education, to be provided for, and to be protected from the adult world of work (Bourdillon, 2006). However, this opinion is very different from the conception of childhood in developing and underdeveloped countries. Children in the research area are seen based on social status or where they are, children from poor families have an obligation to work for their survival or to fulfill family needs. While children from the middle class will be given the opportunity to attend school. Pattanaik argues that social and cultural phenomena such as family systems, modes of production, science and technology, power structures, social class and gender influence the way society constructs the concept of childhood.

Qvortrup outlines the assumptions underlying the structural approach to understanding childhood (Boakye-Boaten, 2010). The first assumption is that childhood is a period in which children function as human and social beings and also as social class categories. This assumption sees childhood as an integral part of social construction. The second is that children influence and are also influenced by changes that occur in society.

This third assumption can be explained by observing the transformation of (traditional) childhood. Children are educated through a life process that is attached to or based on customary rules and traditional and spiritual values. Children are regarded as human beings who need help and direction. In addition, custom, tradition and spiritual components are attached to childcare. However, this conception has changed as the capitalization of rural livelihoods has altered the lifestyle of the indigenous people. They are getting worse and having difficulties to survive. In these conditions, children are transformed into agents of the family economy. Capitalist economic practices, for example by building mines and plantations, have proven to absorb a lot of labor from children.

Sociological attention to children has traditionally centered on the realm of socialization, which sees them as passive actors of social forces within a social structure. Not as complete or fully functioning agents in society. Attention to childhood continues to evolve so that the view of the child is no longer as a passive actor in socialization.

At least three premises are established by the sociology of childhood in contemporary child studies (Tabor, 2016). First, children are seen as social actors who are able to adapt and contribute to adult culture or what is called *interpretive reproduction*. *Interpretive* is the ability and creativity children use to make sense of the social world, and *reproduction* is the idea that children not only absorb existing culture but also contribute to the development of that culture. The second premise is that childhood is socially constructed, hence the diverse concepts of childhood. The third premise moves from studying individual children to studying children’s relationships. In this case, the sociology of childhood not only analyzes children’s relationships with peers, but also analyzes children’s relationships with adults. Relationships with adults analyze the power that adults have in controlling social institutions that shape children’s behavior. In these power relations, children are in a minority position whose role in the social structure is determined by adults. Herland observed parents with problematic parenting that was linked to parents’ perceptions of childhood (Herland, 2018).

In the sociology of childhood (Lee, 1982). First, the pre-industrial notion of children as property or pets, or slaves. Historically children in the pre-industrial period were depicted with examples of neglect, abuse and exploitation. Children as property were investments, as labor, or they were schooled and married off for the economic benefit of future parents. Education and training are means to increase the value of the investment. Children in all classes, excluding the aristocratic class, left home at the age of seven to live with employers or relatives. Children in the pre-industrial paradigm were not autonomous over their own bodies, including sexuality. Adults have power in matters of sexuality involving children. But for pre-industrial societies this was not a mistake because children were part of every aspect of life.

### 5.3 Livelihood resource utilization as a strategy in preventing and remediating child labor in rural South Sulawesi

The sustainable livelihoods approach (SLA) works by understanding the diverse factors within and outside the value chain that contribute to child labor. Child labor is commonly caused by poverty, household characteristics/traditions, parental education and employment, lack of access to education and labor market services, and socio-cultural background.

In addition to identifying the causes, SLA also analyzes what potential can be utilized to improve livelihoods. Poverty generally occurs due to culture and structural pressures. Culture is the socio-cultural background of the community that contributes to and facilitates poverty. For example, the tradition of child marriage, lazy behavior, and closedness to change. Structural pressure can be understood in the context of pressure from rules both sourced from the state and those agreed upon collectively (both written and unwritten). For example, regulations on land tenure, land annexation for the benefit of certain groups, expensive agricultural production costs, and so on. However, cultural and structural poverty cannot be separated; they are interrelated and sometimes difficult to distinguish.

Within the community, there are various components that can be utilized as strategies for preventing and handling child labor. These include social capital, natural capital and human capital. Social capital is an important resource for creating or shaping a new social order. **Social capital** arises from the idea that humans cannot limit the problems faced individually, cooperation and togetherness are needed. The concept of social capital was first introduced by Hanifan (Santoso, 2020), the social capital in question is not in the form of money but togetherness to form a close-knit social group. Bourdieu stated that social structure and function can only be understood through social capital (Coleman, 1988) states that social capital is productive, which plays a role in creating human capital.

In the activities to prevent and handle child labor, the parties have the same view that child labor will have a negative impact on children, so the parties agree to prevent and handle child labor. To build a common view of the impact of child labor, government institutions at the local level and informal and non-formal institutions have an important role. Through a persuasive approach, the internalization of child protection values can work well.

Solidarity and social sensitivity are evident in the research area in various activities. Solidarity can be seen in agricultural activities, deliberations, cleaning competitions, and cultural activities. Solidarity is the initial capital in preventing and handling child labor, therefore solidarity should be directed toward an attitude of care and concern for the future of children. For example, by building agreements related to limiting children’s working time and hours, stopping child marriage, and so on. While social sensitivity is seen in sharing activities, families with sufficient economy tend to help poorer families. For example, prioritizing the use of services from poor families in agricultural activities is done out of empathy.

To ensure that prevention and response work is well coordinated, a group should be formed or the task should be assigned to an existing group such as a traditional institution, youth organization, majelis ta’lim, and so on. This group is tasked with ensuring that prevention and handling actions continue to run with a variety of approaches.

In planning the remediation, community elements and families of child laborers jointly agree on the remediation plan. The families put their trust in the proposed remediation plan. Remediation also provides space for various parties to provide support in accordance with the interests of children, support is provided on the basis of solidarity in the community. Schools will provide educational support for children who drop out of school, employers can provide training and capital assistance, and the government facilitates the various needs of children to be able to escape from child labor status.

**Natural capital**, in the form of land, rivers, swamps-lakes, and forest areas can be managed and utilized to improve community welfare. Utilization of water resources such as rivers and springs for consumption, agriculture, and fisheries; utilization of forests for firewood, collecting sap water, and recreational areas. However, the utilization of natural capital is not fully felt by the community. Inequality in access is a major problem. The control and monopolization of natural resources is a factor that causes economic downturn, because the distribution of benefits is only concentrated on certain people. Equalizing and protecting access to livelihoods is a prerequisite for ensuring equitable distribution of benefits.

In the three research areas, there is no monopoly on access to livelihood sources. Rivers, lakes, swamps, springs can be accessed by all communities with supervision from the local government. Except in Samangki Village, there are conflicts between the Bulusaraung National Park management and the community. Some areas controlled by the community are unilaterally designated as National Park areas, and prohibit community activities in the area.

**Human capital** is the expertise or skills possessed by individuals in implementing livelihood strategies. Human capital is a key requirement for individuals in livelihood. Livelihood strategies rely heavily on human capital (skills and physical health).

**Economic capital** is capital in the form of financial and other economic assets such as cash, loans, means of production, precious metals. The utilization of economic capital is closely related to livelihood strategies. Financial institutions can be a channel for the community to fulfill financial needs such as loans for business/agricultural capital, savings. These activities do not only utilize formal financial institution channels, borrowing and saving activities can be done independently or take place within kinship ties, or group ties such as farmer groups. To maximize the utilization of economic capital, it can be done by forming savings and loan groups, cooperatives, and arisan. Farmers in Africa maximize the utilization of economic capital with the *Village Saving and Loan Association* (VSLA) model. Savings and loan groups use an inclusive approach and aim to create economic resilience for farming households.

The utilization of these four capitals can be implemented in child labor monitoring and remediation activities. Social capital relates to social networks, trust, and empathy (social sensitivity). Natural capital is the potential that can be utilized in livelihoods, economic capital can be utilized as a means to create economic resilience, and human capital is expertise and skills.

**Figure 3:**
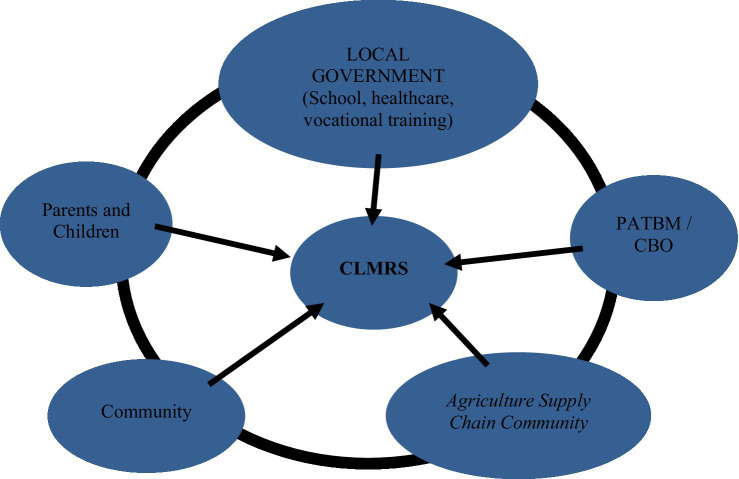
System framework for monitoring and remediation of child labor in rural areas. Source: CLM-ILO and research data (processed).

Child labor monitoring and remediation is a child labor monitoring system that seeks to prevent and address child labor. This concept was adapted from Child Labor Monitoring initiated by the ILO. The child labor monitoring system was first introduced by the ILO in 1990 as a tool to identify and monitor formal workplaces where child labor is present in the Bangladesh Garment Industry. According to the ILO (2005), CLM involves the development of a coordinated, multi-sector monitoring and monitoring process that aims to cover all children within a given geographic area and is closely linked to the enforcement of national child labor laws. The main activity of CLM is to locate child laborers and identify their risks. The CLM then refers the child to service agencies such as education and health, and conducts monitoring to ensure that the child is properly assisted (Figure 3).

**Figure 4:**
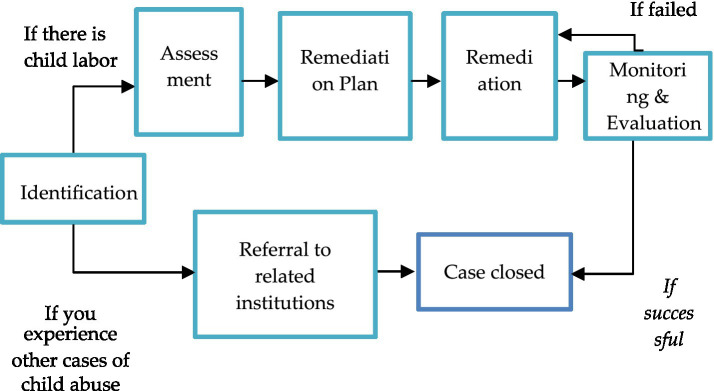
Stages of monitoring and remediation of child labor. Source: Research data (processed).

Monitoring and remediation of child labor is carried out in several stages, namely (Figure 4).

Monitoring and remediation of child labor can also be understood as a voluntary social audit carried out by community groups whose members have a responsibility for the welfare of children. The involvement of other parties is also key to the success of monitoring and remediation of child labor through the distribution of tasks according to their respective capacities, meaning that each party can take part in each stage. All parties can play a role in monitoring and at the same time play a role in remediation activities. If they find a child who is indicated as a child laborer, then they should report it to the local authority for further action. The government and its service institutions such as schools, vocational training centers, health facilities, etc. can serve as referral institutions for remediation. The business world can play a role in remediation such as financial support for poor families, scholarships, and carrying out capacity building to prepare children to enter the workforce according to their age, including building a commitment not to engage in child labor practices.

## 6 Concluding remarks

This research seeks to find appropriate methods in the prevention and remediation of child labor in rural areas. Many previous studies have tried to analyze child labor cases, but this research is different because it uses the SLA approach with a GESI perspective to analyze and understand life experiences, livelihood issues, and examine the relationship between communities and various dimensions such as ecology, politics, and economics. SLA and GESI place the community as an active subject in research, because so far the community, especially vulnerable groups, have been excluded from discussions about living space and livelihoods.

The SLA approach works by understanding the various factors within and outside the value chain that contribute to child labor. Child labor is generally caused by poverty, household characteristics, tradition/culture, parental education and employment, lack of access to education and labor market services, and socio-cultural background. In addition to identifying the causes, the SLA also analyzes what potential can be utilized to improve livelihoods. Within the community, there are various components that can be utilized as strategies to prevent and handle child labor. These include social capital, natural capital, financial capital and human capital. The utilization of these four capitals can be implemented in child labor monitoring and remediation activities. Social capital relates to social networks, trust, and empathy (social sensitivity). Natural capital is the potential that can be utilized in livelihoods, economic capital can be utilized as a means to create economic resilience, and human capital is expertise and skills.

## Data Availability

The raw data supporting the conclusions of this article will be made available by the authors, without undue reservation.
